# Political Identity Over Personal Impact: Early U.S. Reactions to the COVID-19 Pandemic

**DOI:** 10.3389/fpsyg.2021.607639

**Published:** 2021-03-23

**Authors:** Robert N. Collins, David R. Mandel, Sarah S. Schywiola

**Affiliations:** ^1^Toronto Research Centre, Defence Research and Development Canada, Department of National Defence, Government of Canada, Toronto, ON, Canada; ^2^Department of Psychology, University of Waterloo, Waterloo, ON, Canada

**Keywords:** COVID, pandemic, political identity, attitude, belief, polarization, personal impact

## Abstract

Research suggests political identity has strong influence over individuals’ attitudes and beliefs, which in turn can affect their behavior. Likewise, firsthand experience with an issue can also affect attitudes and beliefs. A large (*N* = 6,383) survey (Pew Research and Ipsos W64) of Americans was analyzed to investigate the effects of both political identity (i.e., Democrat or Republican) and personal impact (i.e., whether they suffered job or income loss) on individuals’ reactions to the COVID-19 pandemic. Results show that political identity and personal impact influenced the American public’s attitudes about and response to COVID-19. Consistent with prior research, political identity exerted a strong influence on self-reports of emotional distress, threat perception, discomfort with exposure, support for restrictions, and perception of under/overreaction by individuals and institutions. The difference between Democrats and Republican responses were consistent with their normative value differences and with the contemporary partisan messaging. Personal impact exerted a comparatively weaker influence on reported emotional distress and threat perception. Both factors had a weak influence on appraisal of individual and government responses. The dominating influence of political identity carried over into the bivariate relations among these self-reported attitudes and responses. In particular, the appraisal of government response divided along party lines, tied to opposing views of whether there has been over- or under-reaction to the pandemic. The dominance of political identity has important implications for crisis management and reflects the influence of normative value differences between the parties, partisan messaging on the pandemic, and polarization in American politics.

## Introduction

Amidst a polarized political climate (Jacobson, 2013; Doherty, 2014; Hare and Poole, 2014; Dunlap et al., 2016; Garimella and Weber, 2017), the coronavirus disease 2019 (COVID-19) pandemic has swept across the United States (US). As of the 24th of August 2020, the US reported over 5.5 million cases and 175,000 deaths due to COVID-19 (Centers for Disease Control and Prevention, 2020). The impact of the pandemic is widespread, felt not only in terms of lives lost but also in terms of psychological harm (Centers for Disease Control and Prevention, 2020; Cullen et al., 2020; Serafini et al., 2020) and economic loss (Baker et al., 2020; Fernandes, 2020; Soucheray, 2020), with 20.6 million lost jobs in the US through the early months of the pandemic. The widespread impact of the pandemic has placed it in direct competition with partisan messaging and political identity in shaping the public’s attitudes toward COVID-19 and appropriate response measures.

The COVID-19 pandemic poses unique and difficult management challenges. The disease, caused by severe acute respiratory syndrome coronavirus 2 (SARS-CoV-2; Coronaviridae Study Group of the International Committee on Taxonomy of Viruses, 2020), produces several flu-like symptoms, such as coughing (often with sputum), shortness of breath, muscle aches, and fevers (Centers for Disease Control and Prevention, 2020). Typically, the most acute and deadly symptoms are the associated respiratory illnesses (World Health Organization [WHO], 2020), especially prevalent in older populations and those with compromised immune systems. Like other flu viruses, these respiratory symptoms are also its primary means of transmission, spreading primarily through droplets expelled by coughing and sneezing. The combination of factors makes the virus both highly contagious and potentially deadly. Further complicating matters is the possibility of asymptomatic spread (Anguelov et al., 2020; del Rio and Malani, 2020) and the possibility of limited immunity and vulnerability to reinfection (Batisse et al., 2020; Roy, 2020). The combination of factors necessitated a swift response from institutions and organizations under conditions of great uncertainty and accountability pressures.

### Political Identity and Attitudes About COVID-19

Whereas mixed political messaging marked the initial stage of the pandemic, clearer lines were quickly drawn, and polarization of party elites and the masses followed (Hong and Kim, 2016; Jiang et al., 2020). Much of the divide concerns the perceived threat of COVID-19 and the purported costs and benefits of its management. Specifically, there is a divide over the implementation and extent of response measures such as mask wearing, social distancing, and lockdown procedures. The Democratic Party emphasized the threat of the virus (Pickup et al., 2020) and the potential benefits of broad restrictions—namely, lower cases, transmission, and deaths (Best and Boice, 2020; de Bruin et al., 2020; Hsiang et al., 2020)—as exceeding the economic costs (Green et al., 2020). By comparison, the Republican Party de-emphasized the threat of the virus (Pickup et al., 2020) and highlighted the potential cost of broad restrictions—such as job loss, psychological harm, and delayed treatment of non-COVID related illnesses (Baldwin and Weder, 2020; McKee and Stuckler, 2020)—as outweighing the benefits of broad restrictions (Atlas et al., 2020).

#### Normative Value Differences

The relationship between attitudes, beliefs and political identity is complex. Individual differences in values or biases, such as negativity biases (Hibbing et al., 2014; Lilienfeld and Latzman, 2014), may drive the development of political identity, or people may also be encouraged to adopt values preferred by their ingroup (Tajfel and Turner, 1979; Huddy and Bankert, 2017). Of particular note given the pathogenic salience of the COVID-19 pandemic is the relationship between the “behavioral immune system,” a postulated set of behavioral adaptations which mitigate disease severity, and political conservatism (Schaller and Park, 2011; for meta-analysis see Terrizzi et al., 2013). A strong behavioral immune system, hallmarked by disgust sensitivity and a strong ingroup preference, is positively associated with conservatism. However, pandemic mitigation strategies place this preference in direct conflict with aforementioned Republican messaging and normative values that emphasize individual freedom, government non-intervention, and economic costs.

Another factor to consider is research suggesting conservatism is associated with subjective perceptions of threat (Jost et al., 2003, 2017), particularly as it pertains to mortality salience. Subjective perceptions of threat, real or imagined, can produce emotional distress or, if the threat is imagined or overblown, anxiety (American Psychiatric Association, 2013). Likewise, a longstanding finding is that anxiety is associated with enhanced orienting to perceived threats (Posner, 1980; Bar-Haim et al., 2007; Cisler et al., 2009), further suggesting the two experiences are closely related. Importantly, the research does not imply that Republicans ought to perceive COVID-19 to be a greater threat than Democrats, nor does it predict they ought to experience greater anxiety. It does suggest, however, that to the extent they do perceive threat or experience emotional distress, they ought to be more strongly motivate to mitigate that fear than Democrats. Combined with normative emphasis on individuality and personal freedoms, Republicans support for various COVID-19 mitigation measures be strongly related to personal, subjective assessment of the threat posed by the pandemic.

#### The Role of Partisan Messaging

Regardless of how individuals arrive at their political identity, however, partisan messaging can strongly affect subsequent attitudes and beliefs of affiliated persons (Cohen, 2003; Ward and Tavits, 2019; Pennycook et al., 2020). Even issues that initially seem to lack partisan content often divide along partisan lines. Indeed, political identity plays an obvious and powerful role in shaping the beliefs attitudes of the public (Tajfel and Turner, 1979; Huddy, 2001; Van Bavel and Pereira, 2018; Iyengar et al., 2019; Ward and Tavits, 2019). The attitudes tied to these beliefs frequently become more entrenched over time, creating a feedback loop that increases polarization among both party elites and the public.

Unsurprisingly, research shows that public opinion about COVID-19 has likewise polarized along political party lines (Allcott et al., 2020; Calvillo et al., 2020; Jiang et al., 2020), reflected both offline and in social media analysis (Panda et al., 2020). The views of the public have aligned with worries about the virus and economy espoused by Democrats and Republicans, respectively. An ABC News/Ipsos poll conducted in early May, 2020 revealed that Republicans and Democrats have opposing views on the opening of the economy, with 35% versus 92% respectively opposing an immediate re-opening (Soucheray, 2020). These results are aligned with a separate poll conducted in the same month by CNBC/Change Research in which 97% of Democrats compared to 39% of Republicans were significantly worried about the virus (Pramuk, 2020).

The alignment of individual attitudes with partisan identity posed challenges for its management. Research suggests political identity may influence willingness to engage in ostensibly risky behavior (Makridis and Rothwell, 2020; van Holm et al., 2020) as well as willingness to respect and adhere to policies surrounding management of the virus (Allcott et al., 2020; Brzezinski et al., 2020). This poses a problem for the effective disaster management (Baum, 2011; Gregory, 2016). Understanding how political identity shaped early perceptions of and reactions to COVID-19 is therefore of use to future pandemic management efforts.

### Personal Experience in Attitude Formation

It seems both intuitive and uncontroversial to state that firsthand experience with an event or issue can alter ones’ beliefs and attitudes about that event or issue. The significant spread of COVID-19, even as early as March of 2020 (Centers for Disease Control and Prevention, 2020) affected many individuals and families across the US, both in terms of health effects and job loss. One might expect that individuals personally affected by the pandemic would react differently and develop different attitudes regarding the appropriate response. For instance, we might expect that individuals who suffered personally from COVID-19 would report more negative emotions and greater COVID-19-related threat perceptions than individuals who were not personally affected. Personal experience may even be strong enough to override or neutralize the effects of political identity (Bernstein, 2005; Strauss, 2009; Bernstein and Taylor, 2013; Hersh, 2013).

Indeed, personal experience with crises can affect political identity and participation. For instance, research suggests personal experience of poverty can influence belief about the government’s responsibilities (Noone et al., 2012). Victimization in violent crime can influence political participation and attitudes regarding the justice system (Bateson, 2012; Hersh, 2013). In a similar vein, experience with environmental disasters plausibly linked to climate change can influence attitudes regarding institutional responsibility for climate change (Akerlof et al., 2013; Myers et al., 2013; Unsworth and Fielding, 2014). However, much like facts, personal experience may not always be sufficient to shift deeply held ideological beliefs or political identity (Ogunbode et al., 2017).

### Purpose and Hypotheses

Critically, we know of no study that has directly compared the effects of political identity and personal experience in shaping attitudes and beliefs regarding a crisis. Populations directly affected by crises are rarely large enough, diverse enough, or random enough to make such comparisons. However, increasing polarization combined with the COVID-19 pandemic has created a substantial cross-section of individuals affiliated with both major US political parties who either report having or not having been directly affected by the pandemic. These individuals are distributed over many states and a large geographical area. This unique combination of factors provides an effective means for directly comparing the effects of political identity and personal experience on psychological responses to the COVID-19 pandemic.

To examine how political identity and personal impact (i.e., job or income loss)^1^ shaped early attitudes about COVID-19, we examined the US public’s early reactions as a factor of both political identity and personal impact. We used publicly available data from the Pew Research Center in consultation with Ipsos. We were interested in whether and to what extent each factor influenced individuals’ emotional distress, perceptions of pandemic threat, behavioral responses to the pandemic, support for restrictions, and assessments of the government responses to the pandemic. We were also interested in comparing the effect size of each factor, and whether one was markedly stronger than another. Our primary set of hypotheses held that that both political identity and personal impact play a role in shaping perceptions of and reactions to the COVID-19 pandemic, as well as the relationship between perceptions and reactions. However, we hypothesize the effects of political identity will be more consistent and larger than personal impact across a range of attitudes and responses. Broadly, we predicted that both Democrats and those personally impacted by the pandemic would exhibit greater emotional threat responses, discomfort, support for restrictions, and evaluate the government response more poorly. These results would reflect a rational response to personal impact, and also be consistent with both normative differences in partisan values as well as partisan messaging on the topic.

As a second aim, we also examined how the relationship between attitudes about COVID-19 differed as a function of both personal impact and political identity. We hypothesized that both political identity and personal impact would influence the relationship between emotional distress, perceptions of threat, behavioral responses to the pandemic, support for restrictions, and assessment of the government’s response. However, in line with our earlier hypothesis about main effects, we predicted that political identity would have a larger effect. Specifically, we predict that Republicans’ emotional distress and threat perception will be more strongly correlated with each other and with behavioral discomfort, support for restrictions, and evaluation of government response. Furthermore, because of the differing normative values between Democrats and Republicans, we predict that the relationships between our variables will be defined by perceptions of government under-reaction and over-reaction, respectively.

## Materials and Methods

### Survey Data

We used the Wave 64 survey developed by the Pew Research Center in consultation with Ipsos. The survey was conducted between March 19 and March 24, 2020. The survey contains a representative sample of the US population totaling 11,537 participants: 45% male and 55% female; 11.2% of participants aged 18–29, 32.9% aged 30–39, 30.0% aged 50–64, and 25.9% aged 65+. A majority (55.4%) of participants were college graduates or at a higher educational level, 29.9% had some college experience, and 14.6% had at most a high school degree. The dataset and full documentation on data-collection procedures are available online from the Pew Research Center (Pew Research Center, 2020).

### Grouping Variables

We created two grouping variables to contrast group-level perceptions of COVID-19. The grouping variables were based on Pew survey questions regarding their political affiliation or leaning and whether participants had been affected by the pandemic.

Political identity was measured by asking participants, “In politics today, do you consider yourself a”: (a) “Republican,” (b) “Democrat,” (c) “Independent,” and (d) “Something else.” Participants who answered (c) or (d) were asked a follow-up question, “As of today do you lean more to…” the Republicans or to the Democrats. To avoid ambiguity in the interpretation of political identity, we opted to include only those respondents who answered (a) or (b) to the initial question, excluding individuals who identified as independents or merely leaning toward one party or another.

Personal impact was measured by asking participants, “For each of the following, indicate whether or not is it something that happened to YOU OR SOMEONE IN YOUR HOUSEHOLD because of the coronavirus outbreak” (a) “been laid off or lost a job” and (b) “had to take a cut in pay due to reduced hours or demand for your work.” Participants provided either a “yes” or “no” response to each question. We created a group-level variable by coding participants who responded “no” to both items as 0 and those who responded “yes” to either question or both questions as 1^2^.

### Response Scales

We computed six response scales to measure participants’ perceptions of, and responses to, the COVID-19 pandemic. The scales were based on 36 items from six related, grouped sets of a questions in the Pew survey pertaining to the effect of COVID-19 on participants’: (1) emotional response, (2) threat response, (3) discomfort with various activities, (4) support for government restrictions, (5) evaluation of public response, and (6) evaluation of public under- or over-reaction. These groupings served as the bases for deriving our response scales. To improve the quality of our analyses, we used a combination of manual scale purification and confirmatory factor analyses (CFA) techniques to ensure meaningful interpretation of the results.

The creation of the response scales involved four steps. The first step was scale purification, which involved the *a priori* elimination of items unrelated to our concepts of interest. We re-coded and reverse coded items as needed during this step to achieve consistent ordinal coding of items. The second step was a CFA of the remaining sets of items to ascertain unidimensionality (Flake et al., 2017). We eliminated items with poor factor loading (<0.50) on their respective latent variables during this step. The third step was to assess the invariance of our baseline model across each of our two grouping variables, political identity and personal impact. The fourth step was to derive standardized scores for each of our response scales to use in further analyses.

All CFA were conducted using the *lavaan* package in *rStudio* (Rosseel, 2012). We used polychoric correlations for our ordinal variables (Olsson, 1979; Drasgow, 1986; Holgado-Tello et al., 2010), robust diagonally weighted least sum for our estimator, and the bounds constrained quasi-Newton optimization method. Our criteria for good model fit was a significant χ^2^ test of fit, a comparative fit index (CFI) ≥ 0.90, a Tucker Lewis index (TLI) ≥ 0.95, a root mean square error of approximation (RMSEA) ≤ 0.08, and a standardized root mean square residual (SRMR) ≤ 0.08. The reliability of the response scales was ascertained using ω*_*t*_* (McDonald, 1999; Revelle and Condon, 2019).

To test for invariance across each of our two grouping variables, we followed the four-step approach recommended by Bowen and Masa (2015) for ordinal items, with the addition of a strict invariance test. We first ascertained that the configural model provided a good fit. We then compared it to a weak factorial (or metric) model where factor loadings were constrained to be equal across groups (in addition to the model configuration); next, a strong factorial (or scalar) invariance model where the factor thresholds were constrained to be equal across groups (in addition to factor loadings and the model configuration); finally, a strict (or uniqueness) invariance model where the residuals were constrained to be equal across groups (in addition to factor thresholds, loadings, and model configuration). Because the large sample makes significant χ^2^ difference test results trivial, we accepted the alternative hypothesis of non-invariance if two conditions are met: a significant χ^2^ difference test (Satorra and Bentler, 2010) and a significant decrement in critical model fit indices for nested, more restricted models (either ΔCFI < –0.010 or ΔRMSEA < –0.010; Cheung and Rensvold, 2002; Chen, 2007; Meade et al., 2008; Putnick and Bornstein, 2016).

Test results for χ^2^ and fit indices for each of our CFA models are shown in Table 1. Factor loadings and reliability measures for our initial model are shown in Table 2. Factor loadings and reliability measures for the baseline model (at the end of step 2) are shown in Table 3. Results of the invariance tests are shown in Table 4. Following the elimination of items with poor factor loadings in step 2, the final baseline six response scale model provided a good and reliable fit for the data. Invariance tests revealed the configural models provided a good fit for the data. Invariance tests unambiguously supported the hypothesis of strict invariance for the personal impact grouping variable, with neither a significant χ^2^ nor a significant decrement in model fit indices. For political identity increasing invariance restrictions produced significant differences in χ^2^ values at each step, providing some evidence for non-invariance across the grouping variable. However, our restricted models did not significantly worsen the fit according to either our ΔCFI or ΔRMSEA criterion. The strict invariance criterion also had no effect whatsoever on the fit. Given these results, and given that we were interested in comparisons across both of our primary grouping variables, we proceeded with our unrestricted baseline model for further analysis.

**TABLE 1:** Unidimensionality and reliability analyses for the 6 response scale model.

	Goodness of Fit Test			Fit Indices			
Model	χ *2*	*df*	*p*	CFI	TLI	RMSEA (90% CI)	SRMR
Initial	8474.58	260	<0.001	0.903	0.903	0.070 (0.069,0.072)	0.093
Baseline	2771.39	194	<0.001	0.967	0.967	0.046 (0.044,0.047)	0.056
**Invariance by identity**							
*Configural*	2899.62	388	<0.001	0.971	0.966	0.045 (0.044,0.047)	0.063
*Metric*	3218.68	404	<0.001	0.968	0.963	0.047 (0.045,0.048)	0.070
*Scalar*	3356.46	415	<0.001	0.966	0.962	0.047 (0.046,0.049)	0.064
*Strict*	3356.46	415	<0.001	0.966	0.962	0.047 (0.046,0.049)	0.064
**Invariance by Impact**							
*Configural*	2913.29	388	<0.001	0.973	0.968	0.045 (0.044,0.047)	0.058
*Metric*	2754.90	404	<0.001	0.975	0.971	0.043 (0.041,0.044)	0.058
*Scalar*	2941.40	415	<0.001	0.973	0.970	0.044 (0.042,0.045)	0.058
*Strict*	2941.40	415	<0.001	0.973	0.970	0.044 (0.042,0.045)	0.058

**TABLE 2:** Factor reliability and loadings for the initial six response scale model.

Factor	ω *_*t*_*	Item	Std. Estimate	*SE*	*z*	*p* (>| *z*|)
EMOTION	0.95	a	0.809	0.010	84.58	<0.001
		b	0.853	0.010	89.02	<0.001
		c	0.667	0.012	58.02	<0.001
		d	0.459	0.014	32.53	<0.001
THREAT	0.61	a	0.923	0.013	68.55	<0.001
		b	0.673	0.013	51.59	<0.001
		c	0.532	0.024	21.98	<0.001
EXPOSURE	0.93	a	0.711	0.011	62.71	<0.001
		b	0.960	0.008	113.87	<0.001
		c	0.915	0.015	62.74	<0.001
		d	0.781	0.010	74.82	<0.001
		e	0.796	0.010	78.30	<0.001
RESTRICTION	0.92	a	0.425	0.031	13.66	<0.001
		b	0.864	0.008	115.00	<0.001
		c	0.937	0.008	113.04	<0.001
		d	0.934	0.009	98.37	<0.001
		e	0.914	0.010	91.63	<0.001
		f	0.968	0.006	151.63	<0.001
		g	0.695	0.012	59.93	<0.001
RESPQUAL	0.82	a	0.437	0.013	32.68	<0.001
		b	0.822	0.008	96.96	<0.001
		c	0.861	0.009	98.97	<0.001
		d	0.555	0.011	52.36	<0.001
RESPCAL	0.86	a	0.890	0.007	124.92	<0.001
		b	0.839	0.007	119.92	<0.001

**TABLE 3:** Factor reliability and loadings for the final six response scale model.

Factor (ω *_*t*_*)	ω *_*t*_*	Item	Std. Estimate	*SE*	*z*	*p* (>| *z*|)
EMOTION	0.96	a	0.816	0.011	77.04	<0.001
		b	0.867	0.011	81.13	<0.001
		c	0.678	0.012	57.75	<0.001
THREAT	0.62	a	0.923	0.014	68.12	<0.001
		b	0.676	0.013	51.83	<0.001
		c	0.624	0.024	21.56	<0.001
EXPOSURE	0.93	a	0.711	0.011	62.75	<0.001
		b	0.961	0.008	114.13	<0.001
		c	0.916	0.015	62.98	<0.001
		d	0.780	0.010	74.37	<0.001
		e	0.796	0.010	78.03	<0.001
RESTRICTION	0.95	b	0.864	0.008	113.99	<0.001
		c	0.937	0.008	113.02	<0.001
		d	0.933	0.010	97.97	<0.001
		e	0.913	0.010	91.24	<0.001
		f	0.968	0.006	151.57	<0.001
		g	0.690	0.012	59.23	<0.001
RESPQUAL	0.80	b	0.856	0.008	100.70	<0.001
		c	0.867	0.009	99.49	<0.001
		d	0.503	0.011	44.30	<0.001
RESPCAL	0.86	a	0.890	0.007	124.35	<0.001
		b	0.839	0.007	119.83	<0.001

**TABLE 4:** Invariance tests for the final six response scale model.

Invariance	χ*2*	*df*	Δχ*2*	Δ*df*	*p*(Δχ*2*)	CFI	ΔCFI	RMSEA	Δ RMSEA
Political identity									
*Configural*	2448.80	388				0.971		0.045	
*Metric*	2940.63	404	192.36	16	< 0.001	0.968	–0.003	0.047	0.002
*Scalar*	2877.48	415	51.13	11	< 0.001	0.966	–0.001	0.047	0.000
*Strict*	2877.48	415				0.966		0.047	
Personal impact									
*Configural*	2463.79	388				0.973		0.045	
*Metric*	2507.45	404	16.41	16	0.425	0.975	0.002	0.043	–0.002
*Scalar*	2499.41	415	5.90	11	0.880	0.973	–0.002	0.044	0.001
*Strict*	2499.41	415				0.973		0.044	

To calculate scores for our response scales we used the *lavPredict* function to estimate the value of our latent variables, using the Empirical Bayes Modal approach for categorical variables and bounds constrained quasi-Newton optimization.

The emotion scale (EMOTION) concerned participants’ emotional state in the previous week. Participants were asked, “In the past 7 days, how often have you…”: (a) “felt nervous, anxious, or on edge?”, (b) “felt depressed?”, (c) “felt lonely?”, (d) “felt hopeful about the future?”, and (e) “had trouble sleeping?” Participants rated the frequency of the emotional state using a four-point (1–4) scale ranging from “Rarely or none of the time (less than 1 day)” to “Most or all of the time (5–7 days)”. *A priori*, we excluded (e) as it pertained to behavior rather than emotion. We also excluded (d) because of poor factor loading. The final scale included items (a) – (c), loaded on the factor such that higher values indicate greater emotional distress.

The threat scale (THREAT) concerned participants’ perception of the threat level posed by COVID-19 to public health and the national economy and personal health and finance. Specifically, participants were asked, “How much of a threat, if any, is the coronavirus outbreak for…” (a) “the health of the U.S. population as whole,” (b) “your personal health,” (c) “the U.S. economy,” and (d) “your financial situation.” Participants rated the perceived threat as “not a threat” (1), “a minor threat” (2), or “a major threat” (3). *A priori*, we excluded (d) because it was conflated with our personal impact grouping variable. The final scale included items (a) – (c), loaded on the factor such that higher values indicate greater threat.

The exposure scale (EXPOSURE) scale concerned participants’ ratings of comfort or discomfort for various activities during the pandemic that might increase their exposure to COVID-19. Participants were asked, “Given the current situation with the coronavirus outbreak, would you feel comfortable or uncomfortable doing each of the following?” (a) “visiting with a close friend or family member at their home,” (b) “eating out in a restaurant,” (c) “attending a crowded party,” (d) “going out the grocery store,” and (e) “going to a polling place to vote.” Participants rated their comfort level as “Comfortable doing this” (1) or “Uncomfortable doing this” (2). The final scale included all items, loaded on the factor such that higher values indicate greater discomfort with exposure.

The restriction scale (RESTRICTION) concerned participants’ perceptions of the necessity or non-necessity of various restrictions during the pandemic aimed at curbing the spread of the virus. Participants were asked, “Thinking about some steps that have been announced in some area to address the coronavirus outbreak, in general do you think each of the following have been necessary or unnecessary?” (a) “restricting international travel to the U.S.,” (b) “requiring most businesses other than grocery stores and pharmacies to close,” (c) “asking people to avoid gathering in groups of more than 10,” (d) “canceling major sports and entertainment events,” (e) “closing K-12 schools,” (f) “limiting restaurants to carry-out only,” and (g) “postponing upcoming state primary elections.” Participants rated the necessity of restrictions as “unnecessary” (1) or “necessary” (2). We excluded (a) due to poor factor loading. The final scale included items (b) – (g), loaded on the factor such that higher values indicate greater support for restriction measures.

The government response quality scale (RESPQUAL) concerned participants’ ratings of how the government responded to the COVID-19 pandemic. Participants were asked, “How would you rate the job each of the following is doing responding to the coronavirus outbreak?” (a) “Donald Trump,” (b) “your state elected officials,” (c) “your local elected officials,” (d) “public health officials such as those at the CDC (Centers for Disease and Prevention),” (e) “ordinary people in your community,” and (f) “the news media.” Participants rated the response as “excellent” (1), “good” (2), or “only fair” (3). *A priori*, we excluded items (e) and (f) because they did not pertain to government response. We further excluded (a) due to poor factor loading^3^. The final response scale included items (b) – (d), loaded on the factor such that higher values indicate greater disapproval of the government’s response.

The government response calibration scale (RESPCAL) concerned participants’ perceptions of how well calibrated the government’s response to COVID-19 was. Participants were asked, “Now, thinking about how different people and groups are reacting to the coronavirus outbreak, how would you say each of the following is reacting?” (a) “your state government,” (b), “your local government,” (c) “your local school system,” (d) “ordinary people in your community,” (e) “ordinary people,” and (f) “the people in your household”^4^. Participants rated the reactions as “overreacting to the outbreak” (–1), “reacting about right” (0), or “not taking the outbreak seriously enough” (1). *A priori*, we excluded items (c) – (f) because they did not pertain to the government’s COVID-19 response. The final response scale included items (a) and (b), loaded on the factor such that lower values are associated with perceptions of overreacting and higher values are associated with perceptions of underreacting.

### Statistical Procedure

We excluded from analyses participants who responded (c) or (d) to the political identity grouping variable as well as those that did not provide a complete set of responses for our grouping variables and scales. The final sample included 6,383 respondents, comprised of 1,866 not impacted Republicans, 723 impacted Republicans, 2,569 not impacted Democrats, and 1,225 impacted Democrats.

## Results

### Effect of Political Identity and Personal Impact on Psychological Responses

**FIGURE 1:**
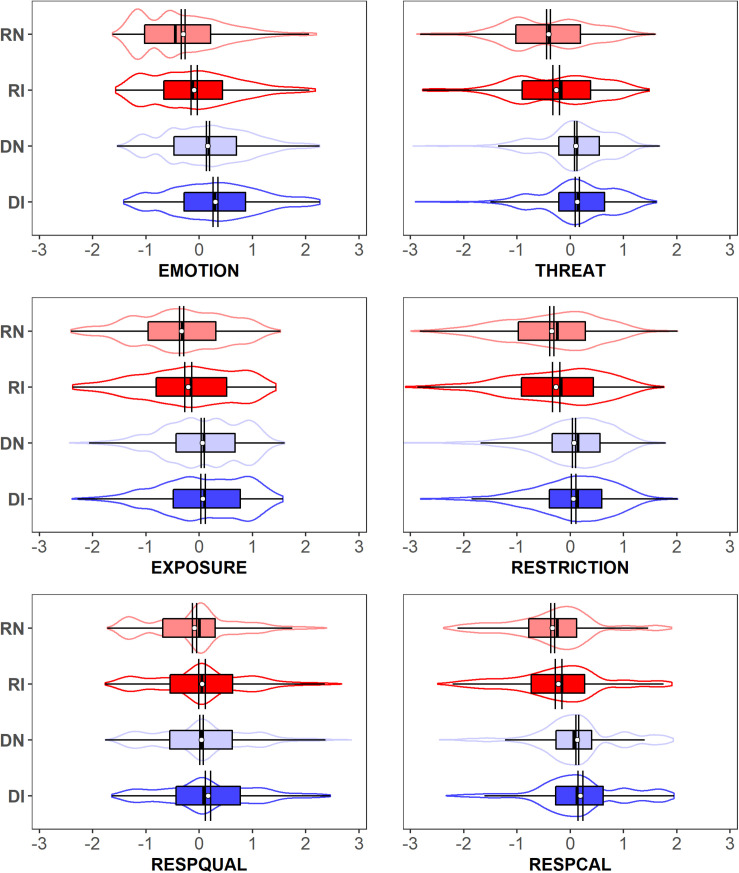
Distribution, means, and variance by political identity (R, Republican; D, Democrat) and personal impact (N, not impacted, I, impacted). Mean and variance are represented by a combination of a point and error bars (95% CIs); sample distribution represented by combining discretized violin plot and a box and whiskers plot.

**FIGURE 2:**
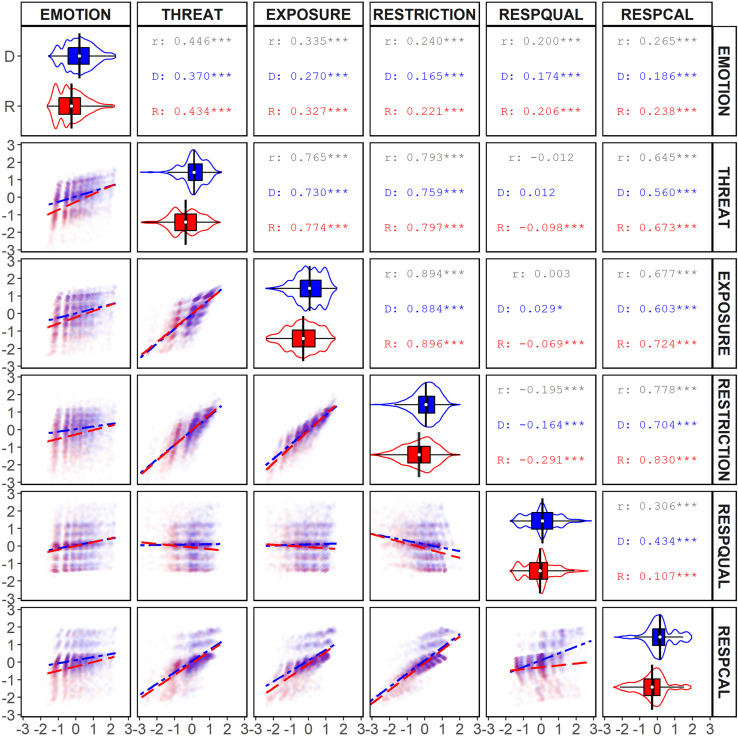
Bivariate analyses of response scales for survey respondents by political identity. The upper-right panels (*R*, Republican; D, Democrat) display the *r*^2^ and significance values (*p < 0.10; ***p < 0.001) for each correlation. The diagonal displays the distribution, means, and 95% CI of responses on each scale. The lower-left panels display scatter plots and correlation lines for each combination of scales (long-dash = Democrat; dot-dash = Republican), with size tracking the density of responses.

**FIGURE 3:**
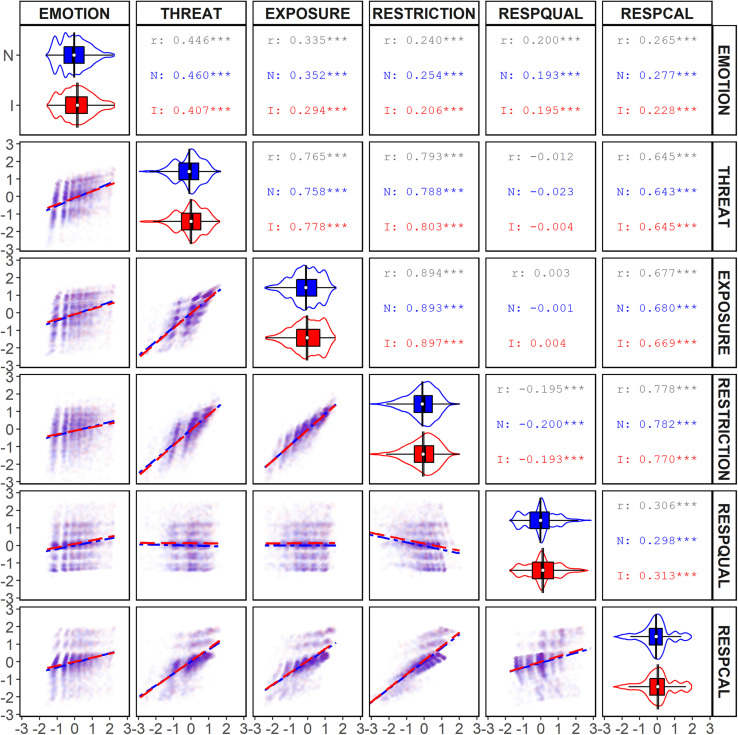
Bivariate analyses of response scales for survey respondents by personal impact. The upper-right panels (*N*, not impacted; I, impacted) display the *r*^2^ and significance values (*p < 0.10; ***p < 0.001) for each correlation. The diagonal displays the distribution, means, and 95% CI of responses on each scale. The lower-left panels display scatter plots and correlation lines for each combination of scales (long-dash = Not impacted; dot-dash = Impacted), with size tracking the density of responses.

We submitted our six response scales to a 2 (Political Identity) × 2 (Personal Impact) between-subjects MANOVA. We calculated 90% confidence intervals (CI) on the effect size measure, η*_*p*_*^2^, using the method prescribed by Smithson (2003).^5^ Figure 1 shows the distributions, means, and 95% CI by political identity and personal impact for each of our response variables. Table 5 summarizes the multivariate results and Table 6 shows the parameter estimates for the univariate results. Additionally, overall distribution and grand means are plotted in Figure 1, whereas marginal distributions and grand means can be found later in Figure 2 (by political identity) and Figure 3 (by personal impact).

**TABLE 5:** Multivariate effects of affiliation and personal impact on response scales.

Effect	*F*	*df*	*p*	η *_*p*_*^2^ [90% CI]
Intercept	35.96	6, 6374	< 0.001	0.033 [0.025,0.039]
Political Identity	123.42	6, 6374	< 0.001	0.104 [0.092,0.115]
Personal Impact	13.70	6, 6374	< 0.001	0.013 [0.008,0.017]
Interaction	1.91	6, 6374	0.076	0.002 [0.000,0.003]

**TABLE 6:** Univariate parameter estimates for affiliation and personal impact on individual response scales.

Scale	Parameter	*B*	*SE*	*t*	*p*	η *_*p*_*^2^ [90% CI]
EMOTION	Intercept	0.164	0.016	10.48	< 0.001	0.017 [0.012,0.023]
	Political Identity	–0.463	0.024	–19.18	< 0.001	0.055 [0.046,0.064]
	Personal Impact	0.140	0.028	5.06	< 0.001	0.004 [0.002,0.007]
	Interaction	0.065	0.044	1.47	0.141	0.000 [0.000,0.002]
THREAT	Intercept	0.104	0.014	7.26	< 0.001	0.008 [0.005,0.012]
	Political Identity	–0.512	0.022	–23.16	< 0.001	0.078 [0.067,0.088]
	Personal Impact	0.029	0.025	1.17	0.243	0.000 [0.000,0.001]
	Interaction	0.116	0.041	2.86	0.004	0.001 [0.000,0.003]
EXPOSURE	Intercept	0.067	0.016	4.23	< 0.001	0.003 [0.001,0.005]
	Political Identity	–0.398	0.024	–16.35	< 0.001	0.040 [0.033,0.048]
	Personal Impact	0.006	0.028	0.22	0.829	0.000 [0.000,0.000]
	Interaction	0.120	0.045	2.68	0.007	0.001 [0.000,0.003]
RESTRICTION	Intercept	0.070	0.016	4.49	< 0.001	0.003 [0.001,0.006]
	Political Identity	–0.419	0.024	–17.35	< 0.001	0.045 [0.037,0.054]
	Personal Impact	–0.009	0.028	–0.31	0.756	0.000 [0.000,0.001]
	Interaction	0.092	0.044	2.07	0.039	0.001 [0.000,0.002]
RESPQUAL	Intercept	0.046	0.017	2.75	0.006	0.001 [0.000,0.003]
	Political Identity	–0.134	0.026	–5.19	< 0.001	0.004 [0.002,0.007]
	Personal Impact	0.120	0.029	4.08	< 0.001	0.003 [0.001,0.005]
	Interaction	0.024	0.047	0.50	0.619	0.000 [0.000,0.001]
RESPCAL	Intercept	0.132	0.016	8.49	< 0.001	0.011 [0.007,0.016]
	Political Identity	–0.462	0.024	–19.32	< 0.001	0.055 [0.047,0.065]
	Personal Impact	0.058	0.027	2.14	0.032	0.001 [0.000,0.002]
	Interaction	0.053	0.044	1.21	0.228	0.000 [0.000,0.001]

*For the coefficient of Personal Identity, 0 = Democrat, 1 = Republican; for the coefficient of Personal Impact, 0 = Not Impacted, 1 = Impacted.*

As shown in Table 5, there were significant main effects of political identity and personal impact and no interaction. Democrats scored higher on most response scales than did Republicans. Participants personally impacted by the pandemic tended to score higher on the response scales than participants who were not personally impacted. The effect size of political identity was medium-to-large, whereas the effect size of personal impact was small (Cohen, 1988; Miles and Shevlin, 2001). This difference in effect size was significant in that the 90% CI surrounding η*_*p*_*^2,^ do not overlap, indicating that political identity was more predictive of participants’ responses than whether they had been personally affected by COVID-19.

As Table 6 shows, the univariate parameter estimates for each of our response scales revealed similar results. There was a small-to-moderate main effect of political identity for each of our response scales. Compared to Republicans, Democrats were more emotionally distressed (EMOTION), perceived a greater threat (THREAT), were more uncomfortable with exposure (EXPOSURE), supported more risk-mitigating restrictions (RESTRICTIONS), expressed more disapproval with the government’s response (REPQUAL), and thought the government was underreacting (RESPCAL). There was a small main effect of personal impact for three of the scales. Impacted respondents reported experiencing more emotional distress (EMOTION), provided poorer ratings of government response (RESPQUAL), and thought the government was underreacting (RESPCAL). There was also a small interaction effect for three of our response scales, manifesting similarly in each. Specifically, personal impact increased the perceived threat (THREAT), discomfort with exposure (EXPOSURE), and support for risk-mitigating restrictions (RESTRICTIONS) more for Republicans than for Democrats, who were largely insensitive to the effects of personal impact on those same scales. It is noteworthy that the interaction is found exclusively in those scales where the effect of personal impact was not significant. Importantly, like the multivariate analysis, the main effect of political identity accounted for a substantially greater portion of variance in scale responses than did personal impact or the interaction effect, except for RESPQUAL in which η*_*p*_*^2^ was similar for the two main effects.

### Correlational Analysis

We submitted each of our six response scales to three sets of bivariate correlational analyses. The first analysis examined the relations among the scales in the overall sample, the second analysis examined the same relations disaggregated by political identity, and the third analysis instead disaggregated the sample by personal impact.

#### Overall Correlations

The results of the overall bivariate correlational analysis are displayed in Figures 2, 3. Each of our scales was significantly and positively correlated with one another except for the RESPQUAL scale, which was not correlated with either the THREAT scale or the EXPOSURE scale and was negatively correlated with the RESTRICTION scale. The significant correlations involving RESPQUAL were weak-to-moderate, whereas all other significant correlations were moderate-to-strong (Cohen, 1988).

Summarizing the results, participants who exhibited greater emotional distress (EMOTION) also perceived greater threat (THREAT), were more uncomfortable with exposure (EXPOSURE), supported greater restrictions (RESTRICTION), rated the government’s response poorly (RESPQUAL), and thought the government was not taking the pandemic seriously enough (RESPCAL). Participants who perceived a high level of threat (THREAT) also were more uncomfortable with exposure (EXPOSURE), supported greater restrictions (RESTRICTIONS), and thought the government was not taking the pandemic seriously enough (RESPCAL). Those who were more uncomfortable with exposure (EXPOSURE) showed greater support for restrictions (RESTRICTION) and thought the government was not taking the pandemic seriously enough (RESPCAL). Participants who supported more restrictions (RESTRICTION) disapproved of the government’s response (RESPQUAL), and thought the government was not taking the pandemic enough (RESPCAL). Finally, participants’ who rated the government response poorly (REPQUAL) typically thought the government was not taking the pandemic seriously enough (RESPCAL).

#### Correlations by Political Identity

Figure 2 shows the results of the bivariate correlational analysis by political identity, whereas Table 7 shows the results of tests of independent correlations (Cohen et al., 2003) contrasting correlational strength by political identity. Political identity had a significant effect on each of the correlations except for EMOTION and RESPQUAL. In most cases, the difference manifested as a higher *r* for Republicans than for Democrats. This trend was reversed for the correlations between RESPQUAL and each of: THREAT, EXPOSURE, and RESTRICTION; as well as the correlation between RESPQUAL and RESPCAL. There were two particularly interesting findings. First, the correlations between RESPQUAL and both THREAT and RESTRICTION—neither significant in the overall population nor within the Democratic population—were significantly and negatively correlated within the Republican population. Second, the size of the difference in correlation between RESPQUAL and RESPCAL scales was very large. Specifically, the correlation between the two scales was moderate-to-strong for Democrats and weak for Republicans. Democrats who rated government’s response poorly (RESPQUAL) tended to perceive the government as not taking the pandemic seriously enough (RESPCAL). By contrast, the scatterplots reveal a population of Republicans who rated the government’s response poorly (RESPQUAL) because they thought the government was overreacting (RESPCAL). This difference is revealing of how political identities relate assessments of performance to perceptions of seriousness.

**TABLE 7:** Tests of independent correlations contrasting the strength of response scale correlations by *political identity* and *personal impact*.

		Political Identity		Personal Impact		Δ | *z*|	
Scale 1	Scale 2	*z*	*p*	*z*	*p*	*z*	*p*
EMOTION	THREAT	3.35	0.001	3.12	0.002	–0.17	0.867
EMOTION	EXPOSURE	2.72	0.006	3.09	0.002	0.26	0.798
EMOTION	RESTRICTION	2.53	0.011	2.43	0.015	–0.07	0.943
EMOTION	RESPQUAL	1.43	0.152	0.08	0.936	–0.96	0.338
EMOTION	RESPCAL	2.35	0.019	2.49	0.013	0.10	0.924
THREAT	EXPOSURE	4.46	< 0.001	2.3	0.021	–1.53	0.127
THREAT	RESTRICTION	4.21	< 0.001	1.92	0.054	–1.62	0.105
THREAT	RESPQUAL	4.81	< 0.001	0.88	0.378	–2.77	0.006
THREAT	RESPCAL	7.93	< 0.001	0.19	0.848	–5.47	< 0.001
EXPOSURE	RESTRICTION	2.41	0.016	0.81	0.418	–1.13	0.257
EXPOSURE	RESPQUAL	4.28	< 0.001	0.26	0.791	–2.84	0.005
EXPOSURE	RESPCAL	9.48	< 0.001	0.93	0.350	–6.04	< 0.001
RESTRICTION	RESPQUAL	5.86	< 0.001	0.35	0.725	–3.9	< 0.001
RESTRICTION	RESPCAL	13.66	< 0.001	1.42	0.155	–8.66	< 0.001
RESPQUAL	RESPCAL	15.57	< 0.001	0.8	0.426	–10.44	< 0.001

We also examined whether the proportion of variance accounted for between scale responses was similar for Republicans and Democrats. We calculated the *r*^2^ and 90% CIs for each of our correlations using the method prescribed by Smithson (2003; see also Steiger and Fouladi, 1992). The results are displayed in Table 8. In most cases where *r* significantly differed, this was reflected in the *r*^2^ analysis in the sense that *r*^2^ was higher for Republicans and non-overlapping with Democrats. The exception was the RESPCAL by RESPQUAL correlation, in which the *r*^2^ was somewhat higher for Democrats than for Republicans.

**TABLE 8:** Percentage of variance accounted for between response scales, *r*^2^ [90% CI], as a function of political identity.

		Political Identity		
Scale 1	Scale 2	Democrat	Republican	Overlapping
EMOTION	THREAT	0.137 [0.120,0.154]	0.189 [0.166,0.212]	No
EMOTION	EXPOSURE	0.073 [0.060,0.087]	0.107 [0.089,0.127]	No
EMOTION	RESTRICTION	0.027 [0.019,0.036]	0.049 [0.036,0.063]	No
EMOTION	RESPQUAL	0.030 [0.022,0.040]	0.042 [0.031,0.056]	Yes
EMOTION	RESPCAL	0.035 [0.026,0.045]	0.057 [0.043,0.072]	Yes
THREAT	EXPOSURE	0.533 [0.514,0.551]	0.599 [0.579,0.619]	No
THREAT	RESTRICTION	0.576 [0.558,0.593]	0.635 [0.616,0.654]	No
THREAT	RESPQUAL	0.000 [0.000,0.001]	0.010 [0.004,0.017]	No
THREAT	RESPCAL	0.314 [0.294,0.335]	0.453 [0.428,0.476]	No
EXPOSURE	RESTRICTION	0.782 [0.771,0.792]	0.802 [0.790,0.813]	Yes
EXPOSURE	RESPQUAL	0.001 [0.000,0.003]	0.005 [0.001,0.010]	Yes
EXPOSURE	RESPCAL	0.363 [0.343,0.384]	0.524 [0.501,0.545]	No
RESTRICTION	RESPQUAL	0.027 [0.019,0.036]	0.085 [0.068,0.103]	No
RESTRICTION	RESPCAL	0.496 [0.477,0.515]	0.690 [0.672,0.706]	No
RESPQUAL	RESPCAL	0.189 [0.170,0.208]	0.012 [0.006,0.019]	No

#### Correlations by Personal Impact

Figure 3 shows the results of the bivariate correlational analysis by personal impact, whereas Table 7 shows the results of tests of independent correlations contrasting correlational strength by political identity. Personal impact had a significant effect on the correlations between EMOTION and each of: THREAT, EXPOSURE, RESTRICTION, and RESPCAL. In each case, the difference was reflected by a larger *r* for the not-impacted population than for the impacted population. The difference in correlation between THREAT and EXPOSURE was also significant. In this, the difference was characterized by the opposite trend: a lower *r* for the not impacted sample than for the impacted sample.

We also compared the proportion of variance accounted for between scale responses as a function of personal impact. We calculated the *r*^2^ and 90% CIs for each of our correlations (Smithson, 2003; see also Steiger and Fouladi, 1992). The results are displayed in Table 9. Only the *r*^2^ for the significant correlations involving EMOTION and both THREAT and EXPOSURE differed. Like with the analysis of *r, r*^2^ was greater for the not impacted sample than for the impacted sample.

**TABLE 9:** Percentage of variance accounted for between scales, *r*^2^ [90% CI], as a function of personal impact.

		Personal Impact		
**Scale 1**	**Scale 2**	**Not Impacted**	**Impacted**	**Overlapping**
EMOTION	THREAT	0.212 [0.194,0.230]	0.165 [0.140,0.191]	No
EMOTION	EXPOSURE	0.124 [0.109,0.140]	0.086 [0.067,0.107]	No
EMOTION	RESTRICTION	0.065 [0.053,0.077]	0.042 [0.029,0.058]	Yes
EMOTION	RESPQUAL	0.037 [0.029,0.047]	0.038 [0.025,0.053]	Yes
EMOTION	RESPCAL	0.077 [0.065,0.090]	0.052 [0.037,0.069]	Yes
THREAT	EXPOSURE	0.574 [0.558,0.590]	0.605 [0.581,0.627]	Yes
THREAT	RESTRICTION	0.621 [0.606,0.635]	0.645 [0.623,0.665]	Yes
THREAT	RESPQUAL	0.001 [0.000,0.002]	0.000 [0.000,0.001]	Yes
THREAT	RESPCAL	0.413 [0.395,0.432]	0.417 [0.388,0.444]	Yes
EXPOSURE	RESTRICTION	0.798 [0.789,0.807]	0.804 [0.791,0.817]	Yes
EXPOSURE	RESPQUAL	0.000 [0.000,0.000]	0.000 [0.000,0.001]	Yes
EXPOSURE	RESPCAL	0.463 [0.445,0.481]	0.448 [0.420,0.475]	Yes
RESTRICTION	RESPQUAL	0.040 [0.031,0.050]	0.037 [0.024,0.052]	Yes
RESTRICTION	RESPCAL	0.611 [0.596,0.626]	0.593 [0.569,0.616]	Yes
RESPQUAL	RESPCAL	0.089 [0.076,0.102]	0.098 [0.078,0.120]	Yes

#### Comparison of Group Effects on Correlations

The rightmost columns of Table 7 compare the difference in *z* scores by political identity and personal impact using Rosenthal (1991) method. The findings show that political identity had as large or larger an effect than personal impact on all correlations in which the two moderators significantly differed. In no case was the effect of personal impact on correlations between response scales significantly larger than political identity.

## Discussion

The present findings supported the predictions outlined in our primary and secondary hypotheses. Compared to Republicans, Democrats were more emotionally distressed, perceived greater threat, showed greater discomfort with exposure, supported greater restrictions, were pessimistic about the government response, and thought the government was under-reacting. These effects, in turn, were significantly larger than the effects of self-reported personal impact from COVID-19. Supporting the predictions outlined in our secondary hypotheses, political identity also moderated the relations among the responses to a greater extent than personal impact. In fact, personal impact only weakly predicted participants’ responses, achieving significance for only half as many comparisons. Moreover, personal impact had only a small effect on the relations among scale ratings. Finally, the effect size of political identity was clearly larger than personal impact in most cases.

### Dominance of Political Identity

Although the greater relevance of political identity on COVID-related attitudes and beliefs may appear counter-intuitive, the observed partisan split in the current survey closely resembles the partisan divide observed in other research (Pickup et al., 2020). For our measures of personal response to the pandemic, Democrats showed increased emotional distress, threat perception, and discomfort with exposure, all of which are consistent with prior research as well as partisan messaging. Research also shows Democrats report lower levels of happiness or life satisfaction than Republicans (Napier and Jost, 2008; Mandel and Omorogbe, 2014; Wojcik et al., 2015), and that conservatism is correlated with lower perceived virus threat (Calvillo et al., 2020), both providing further context for the empirical result. Regarding opinions on policy and evaluations of government response, greater support for restrictions and perception of under-reaction among Democrats tracked closely with the differences in partisan messaging regarding the topic (Panda et al., 2020; Pickup et al., 2020) as well as the normative value differences of members of the two parties. Democrats tend to show greater support for government and top–down government interventionist strategies (Schlenker et al., 2012).

Further supporting this hypothesis is the correlation between the quality of the government’s responses (RESPQUAL) and the perceived calibration of those responses (RESPCAL). In this case, Republicans were somewhat more likely to rate the quality of responses to the pandemic as poorer if they believed the government was overreacting to the pandemic, producing a negative directional shift in the correlation. In contrast, Democrats were consistent and strongly inclined to rate the quality of responses as poorer if they believed the government was not taking the pandemic seriously enough. These results closely mirror partisan messaging on the topic in which Democrat sources place greater emphasis on the threat of the virus, whereas Republican sources place greater emphasis on balancing economic costs (Pickup et al., 2020). Accordingly, as the findings indicate, Democrats tend to agree in their perceptions of government underreach in pandemic risk mitigation as a basis for poor performance, whereas Republican was less homogeneous as a group in their attribution of poor performance. This pattern reflects the more general tendency of Democrats to place greater value on collective welfare and to offer greater support for government intervention, whereas Republicans tend to place greater value on individualism and are skeptical of government overreach (Schlenker et al., 2012).

### Pandemic Spread, Geographic Distributions, and Political Identity

An alternative hypothesis invokes the geographic progression of the virus. The survey we examined includes data collected in early March, at a time when urban areas—which typically lean Democrat (Scala and Johnson, 2017; Badger, 2019)—and Democrat controlled coastal states were experiencing the brunt of the initial wave. Therefore, one might argue, it is unsurprising that Democrats may report greater levels of emotional distress, threat perception, discomfort with exposure, and support more restrictions. In fact, a Pearson Chi-Square test indicated the proportion of Democrats who were impacted (32.3%) was higher than the proportion Republicans who were impacted (27.9%), *X*^2^(1, *N* = 6 383) = 13.81, *p* < 0.001. However, there are two reasons to doubt this.

First, there are the weak effects of personal impact on the measures examined. Second, when the interaction was significant, Republicans were most sensitive to the effect of impact. Rather, we would argue that partisan messaging and normative value differences between Democrats and Republicans offer a better explanation of the observed differences. Nevertheless, the hypothesis does highlight the need for follow-up research that tracks longitudinal changes in attitudes and beliefs among persons as the geographic makeup of the pandemic evolves.

### Negativity Bias, Emotional Distress, and Threat Motivation

Interestingly, our results seem to conflict with research on negativity bias (Hibbing et al., 2014) and the behavioral immune system (Schaller and Park, 2011; Terrizzi et al., 2013) in conservatives and Republicans. The former would imply Republicans ought to be more pessimistic about the threat posed by a novel, perhaps ambiguous pandemic. The latter would imply Republicans might be quicker to support measures to mitigate the threat and preserve the ingroup. Both predictions run counter to what we have described above. However, we suggest this conclusion is premature for reasons discussed below.

An intriguing finding from the present research is that Republicans exhibited stronger relations among the response measures than Democrats (as measured by *r*^2^), with the exception of the already-discussed RESPQUAL and RESPCAL correlation. For instance, compared to Democrats, Republicans reported emotions that were more strongly related to pandemic-specific evaluations, including the overall threat posed by COVID-19, discomfort engaging in exposure-amplifying behaviors, and the perceived necessity of enforcing pandemic-mitigating restrictions. Reflexively or intuitively, one might attribute these differences to previously discussed normative values: a population of perfectly rational individualist Republicans might be more sensitive to self-relevant experience in their perceptions of appropriate responses. Conversely, a population of perfectly rational collectivist Democrats might place less emphasis on self-relevant experience in their perceptions of appropriate responses. If true, the *r*^2^ would be predictably higher in the former than for the latter. In fact, we see some converging evidence for this in the MANOVA interactions: Republicans were indeed more sensitive to the effect of personal impact when it came to perceived threat of the pandemic (THREAT), discomfort with exposure (EXPOSURE) and their support for pandemic-related mitigation measures (RESTRICTION). Curiously, this identity-specific sensitivity did not extend to evaluations of the government’s response, RESPQUAL and RESPCAL, complicating the interpretation somewhat, though not ruling it out.

A closer examination further reveals that Democrats tended to show less variability in the scales for which large differences in the bivariate correlations were observed. A Levene test of variance heterogeneity confirmed the variability in responses for four of the six scales—THREAT, EXPOSURE, RESTRICTION, and RESPCAL—was greater for Republicans than for Democrats according to the standard error of the mean measure, all *p* < 0.001. The opposite was true for just one of our scales, RESPQUAL, *p* = 0.039, and in this case both main effects were small and the difference in variance was also small. Variance in the EMOTION scale did not differ by political identity, *p* = 0.499. Where greater consensus and lower variability exists in a sample, error (or noise) variability grows as a proportion of total variability between responses, reducing the strength of subsequent correlations. The theoretical cause of differing variance, then, is of particular interest. One potential explanation is a greater majority consensus among Democrats regarding the threat posed to public health and the economy (Pramuk, 2020; Soucheray, 2020), supported by the low sensitivity to personal impact status of Democrats on several response scales; namely, THREAT, EXPOSURE, and RESTRICTION.

Another potential explanation supported by prior research is that conservatives are more fear-motivated than liberals in attitude and belief formation (Jost et al., 2003; Lilienfeld and Latzman, 2014; Jost et al., 2017). Critically, as we pointed out in our hypotheses, this research does not indicate that Republicans ought to feel more threat than Democrats on a particular issue, which itself may depend on subjective perceptions of mortality salience (Jost et al., 2017). However, such research does imply that Republicans’ attitudes and beliefs are more greatly influenced by the experience of fear and the desire to mitigate it. Republicans are not necessarily insensitive to the threat posed by the virus (Lilienfeld and Latzman, 2014), nor the desire to mitigate the spread of the disease and protect the ingroup (Schaller and Park, 2011). However, they may be motivated to engage in cognitively complex reasoning to balance those concerns against fears of government encroachments on personal freedoms (Schlenker et al., 2012), increasing the variability in their responses and strengthening correlations between self-relevant information and subsequent support for pandemic management measures. This notion provides a tenable explanation for the greater *r*^2^ among Republicans. That is, Republicans are responding as we might expect to perceptions of threat posed by the pandemic. Those who feel threatened have a strong desire to mitigate that fear and support restrictions. However, that effect is moderated by the overall lower perception of threat posed by COVID-19 (see Calvillo et al., 2020). Indeed, a Hartigans’ dip test of unimodality (Hartigan and Hartigan, 1985) reveals Republican responses on the THREAT scale are bimodal (*D* = 0.022, *p* < 0.001), regardless of whether they were impacted (*D* = 0.031, *p* < 0.001) or not (*D* = 0.021, *p* < 0.001). Thus, we suggest the interactions and correlational findings do not fundamentally conflict with pre-existing research on the relationship between negativity-bias (Hibbing et al., 2014), the behavioral immune system (Lilienfeld and Latzman, 2014), and political identity.

### Revisiting the Role of Personal Experience

It is worth further consideration just how small a role personal impact, in the form of income or job loss, played in shaping perceptions regarding the COVID-19 pandemic. Personal impact accounted for just 1.3% of variance in multivariate responses and even less for the univariate analysis. Furthermore, personal impact had little influence on the relations among the response scales as well. This contradicts both intuition and some research on the topic (Bateson, 2012; Akerlof et al., 2013; Unsworth and Fielding, 2014), and aligns more closely with opposing research stating personal experience has little effect on values and beliefs (Unnever et al., 2007; Ogunbode et al., 2017). One explanation is the effects of experiential learning are stronger for persons less engaged with the topic (Myers et al., 2013). By contrast, highly engaged individuals used motivated reasoning (Kunda, 1990) and interpretation of facts (Gaines et al., 2007; Meirick, 2013; Kraft et al., 2015) to preserve existing attitudes and beliefs (Myers et al., 2013). The intense media coverage and partisan messaging surrounding the pandemic, however, ensured high engagement for the population, and may buffer attitudes and beliefs against personal impact in the short-term. Alternatively, the effects of the personal impact variable we have analyzed (i.e., job or income loss) may produce lagging effects that were not yet fully realized by the individuals who experienced them. Consequently, a future retrospective study could analyze whether personal experience with the COVID-19 pandemic shaped long-term attitudes or political identity. The salience and severity of the impact, which supported only a coarse analysis in the present study, may also be worth further scrutiny at a finer level.

### Political Identity, Normative Values, and Preferences in Pandemic Response

While our analyses reveal the effect of political identity on pandemic-related emotions and attitudes, the present research is not meant to judge the alternative positions predicted by political orientation from a prescriptive stance. Accordingly, we make no attempt to judge how close Democrats and Republicans come to a “proper reaction” to the pandemic. Our use of scare quotes signals our view that the task of establishing a normative basis for affective and attitudinal response evaluation is a deeply value-laden exercise. It assumes—falsely, we would argue—that there is a single correct reference point from which to judge responses to the pandemic (such as those plans adopted and actions taken by government officials) as well as responses to those responses (such as those representing the attitudes of the public toward the government officials’ plans and actions). Rather, pandemics and the responses they trigger reflect complex value-tradeoffs. Political polarization can obscure these tradeoffs by focusing partisans on the values most important to their own side, while minimizing the importance of “out-group” values or even delegitimizing them. Bridging this divide—such that the effects of partisanship are minimized and all parties negotiate in good faith—is a difficult problem in its own respect, and much research effort has been conducted to identify its underpinnings, complications, and possible solutions (Clinton et al., 2021; Green et al., 2020; Rosenbaum, 2020), should any exist.

In this sense, the COVID-19 pandemic is a stark example of what social policy analysts and planners call a *wicked problem* (Churchman, 1967; Rittel and Webber, 1973). Wicked problems are defined by a set of 10 clearly defined characteristics, but for our purposes could be summarized as: a unique problem that is not well-understood until after it is solved, but that planners only get one chance to solve, that has neither an objectively correct solution nor a clear stopping rule. The COVID-19 pandemic is unlike any we have seen in modern history—potentially more severe than the Spanish Flu of 1918 (Ashton, 2020; Javelle and Raoult, 2020; Petersen et al., 2020)—for which we get one chance to solve. Furthermore, there is no objectively correct solution for its management, but rather societies afflicted by the pandemic face a complex series of trade-offs between values, each of which has their own short-, medium-, and long-term implications to consider (Baldwin and Weder, 2020; Best and Boice, 2020; Fernandes, 2020; Hsiang et al., 2020).

### Conclusion, Limitations, and Future Directions

Rather than prescribe solutions for the COVID-19 pandemic or the crisis of partisanship—neither of which are aims of our inquiry or realistically within our field of expertise—our intention was to compare the size and influence of political identity and personal impact on shaping attitudes and beliefs using a large, well-powered study. We have shown that the effect of political identity looms large over emotions, attitudes, and beliefs related to the COVID-19 pandemic, as well as the relationship among these psychological measures. Counter-intuitively, this effect largely overshadowed the arguably more salient and immediate factor of personal experience with the pandemic.

Critically, however, given the correlational nature of our data, we cannot make definitive claims about causal directionality in the various measures considered in this research. As well, political self-identification can be a poor measure or political ideology, and that values-based questionnaires more accurately index political identity (Greene, 1999; Bankert et al., 2017; Huddy and Bankert, 2017). Nevertheless, the strong effects of self-identified political identity observed here join a growing body of literature regarding partisan effects on pandemic related attitudes and beliefs (Conway et al., 2020; Jørgensen et al., 2020) and the complications this poses for its management (Clinton et al., 2021; Gollwitzer et al., 2020; Green et al., 2020). This suggests the value of further research and consideration of both normative value differences and partisan polarization in crafting effective management of future pandemics.

## Data Availability Statement

Publicly available datasets were analyzed in this study. This data can be found here: https://www.pewresearch.org/politics/dataset/covid-19-late-march-2020/.

## Author Contributions

DM contributed to the primary conceptualization with secondary contributions by RC. RC and SS contributed to primary data analysis. RC contributed to primary data visualization. RC contributed to primary writing with secondary contributions from SS and DM. DM and RC contributed to primary editing and oversight. All the authors contributed to the article and approved the submitted version.

## Conflict of Interest

The authors declare that the research was conducted in the absence of any commercial or financial relationships that could be construed as a potential conflict of interest.

## Footnotes

1 Although we were prescriptively interested in the personal experience of having contracted and experienced COVID-19, this data was not available in the survey data analyzed.

2 We treated this variable as binary rather than additive or ordinal because we were concerned about the response characteristics of the population. Because the question does not distinguish between ‘YOU’ or ‘SOMEONE IN YOUR HOUSEHOLD,’ nor the number of earners in the household, several hypotheticals arise which complicate interpretation. Consider that a two-person household where both adults lost their jobs might answer “yes” to (a) and “no” to (b), whereas a similar household where one adult lost a job and the other lost hours may answer “yes” to both, despite the fact the former household is clearly worse off. Consider also a person may answer “yes” to both questions if they believed losing a job qualified as taking a pay cut, or a single-income family household in which the sole earner lost their job.

3 Despite poor reliability, opinions of the president are obviously relevant to the topic at hand. To that end, we present a brief analysis of the observed variable in Appendix A.

4 There was no item (d) in the survey.

5 90% CI are used for our effect size to ensure the inferences match those of our measures *F* and *t* for the MANOVA and univariate parameter estimates, respectively. For example, it is possible to have a significant main effect despite the 95% confidence intervals for the effect size including 0. The narrower confidence interval is related to the fact that squared effect-size measures like η*_*p*_*^2^ cannot take on negative values (Smithson, 2003).

## Appendix A

A 2 (Political Identity) × 2 (Personal Impact) univariate analysis of variance (ANOVA) on item (a), omitted from the government response quality (RESPQUAL) scale and concerning respondents rated quality of Donald Trump’s response to the pandemic, revealed a main effect of political identity, *F* (1, 6379) = 4544.72, *p* < 0.001, and a significant interaction, *F*(1,6379) = 26.74, *p* < 0.001. The effect of personal impact was not significant, *p* = 0.448. The main effect of political identity was due to greater disapproval of Trump among Democrats (*M* = 3.50, *SE* = 0.01) than among Republicans (*M* = 1.62, *SE* = 0.02). The significant interaction manifested as opposing effects of personal impact for Democrats and Republicans. Impacted Democrats (*M* = 3.42, *SE* = 0.02) were less disapproving of Trump than not-impacted Democrats (*M* = 3.58, *SE* = 0.02), whereas impacted Republicans (*M* = 1.68, *SE* = 0.03) were more disapproving of Trump than not impacted Republicans (*M* = 1.55, *SE* = 0.02). The interaction suggests personal impact buffered opinions of the president: when personally affected, Democrats were slightly more forgiving of the President’s responses, whereas Republicans were slightly less so.
